# An Immunohistochemistry-Based Molecular Subtyping Approach for Capturing Clinical Outcome Heterogeneity in Bladder Cancer

**DOI:** 10.3390/diagnostics16132055

**Published:** 2026-06-30

**Authors:** Yuhan Chen, Lingkai Cai, Xiao Yang, Yiran Tao, Baorui Yuan, Zhengye Tan, Hao Yu, Meiling Bao, Qiang Lu

**Affiliations:** 1 Department of Urology, The First Affiliated Hospital with Nanjing Medical University, Nanjing 210000, China; 2 Department of Pediatric Surgery, Women’s Hospital of Nanjing Medical University (Nanjing Women and Children’s Healthcare Hospital), Nanjing 210000, China; 3 Department of Pathology, The First Affiliated Hospital with Nanjing Medical University, Nanjing 210000, China

**Keywords:** bladder cancer, molecular subtyping, immunohistochemistry

## Abstract

**Backgrounds:** Bladder cancer shows pronounced biological heterogeneity that underlies its variable clinical course and prognosis. Our study aims to delineate clinically relevant differences in bladder cancer using an immunohistochemistry-based molecular subtyping approach. **Methods**: This retrospective study included 590 patients with bladder cancer treated at a single center. Tumors were stratified into luminal versus non-luminal categories according to CK20, GATA3, CK5/6, and CK14. Associations between molecular subtype, histopathological growth patterns, pathological response to neoadjuvant chemotherapy (NAC), and clinical survival endpoints were analyzed. Overall survival (OS), recurrence-free survival (RFS), and progression-free survival (PFS) were evaluated through Kaplan–Meier survival curves together with Cox proportional hazards regression analyses. **Results**: Non-luminal tumors exhibited significantly more aggressive pathological growth patterns, including higher levels of tumor budding (*p* = 0.002), a predominance of non-cohesive or spindle/single-cell architecture (*p* = 0.003), and more frequent disseminated spreading patterns (*p* = 0.001), whereas luminal tumors more commonly displayed a higher frequency of tertiary lymphoid structures (TLSs; *p* = 0.041). Among patients receiving NAC, non-luminal tumors achieved a significantly higher pathological complete response (pCR) rate compared with luminal tumors (*p* = 0.007), while no significant inter-subtype difference was detected in pathological downstaging between subtypes (*p* = 0.126). Despite inferior pathological response, luminal tumors demonstrated significantly improved OS (*p* = 0.003), RFS (*p* = 0.002) and PFS (*p* < 0.001) compared with non-luminal tumors. In multivariable Cox regression analysis, molecular subtype was identified as an independent predictor of OS, with luminal tumors showing a lower mortality risk (HR = 0.51, 95% CI 0.33–0.79, *p* = 0.003). **Conclusions:** These findings indicate that pathological response and long-term survival follow distinct, subtype-dependent trajectories in bladder cancer. Favorable pathological response does not necessarily correspond to improved long-term survival across molecular subtypes.

## 1. Introduction

Bladder cancer represents one of the most frequently diagnosed malignancies within the urologic system worldwide [1,2]. However, the clinical management of bladder cancer is complicated by its pronounced heterogeneity, with marked variability in clinical behavior, treatment response, and patient outcomes [3]. Substantial differences in prognosis are observed even among patients with similar clinicopathological features and disease stage [4]. This pronounced heterogeneity has important clinical implications across multiple treatment settings, underscoring the necessity of investigating the biological basis underlying bladder cancer heterogeneity to improve risk stratification and therapeutic decision-making [5].

In an effort to systematically capture and interpret this biological heterogeneity, multiple transcriptome-based molecular classification systems have been independently developed over the past decade, including the Lund taxonomy, the University of North Carolina (UNC) classification, the MD Anderson Cancer Center classification, and molecular profiling efforts from The Cancer Genome Atlas (TCGA) project [3,6,7,8]. In parallel, an international consensus molecular classification was proposed to reconcile these parallel systems into a unified framework [9]. Despite differences in study design and analytical methodologies, these frameworks have consistently identified highly concordant intrinsic molecular lineages across independent cohorts, most notably the basal and luminal differentiation axis [7,8].

Basal tumors typically show the enrichment of basal/squamous-like markers such as KRT5/6 and KRT14 and exhibit phenotypes associated with a less differentiated, aggressive biological state. In contrast, luminal-type tumors are characterized by urothelial differentiation markers, such as GATA3 and uroplakins and are associated with differentiation programs and often harbor FGFR3 mutations or signaling activation, which are linked to more favorable biological behavior [10]. Increasing lines of evidence indicate that intrinsic molecular subtypes are associated with differential patterns of response to neoadjuvant chemotherapy (NAC) and long-term clinical outcomes, indicating that these classifications reflect biologically and clinically meaningful tumor heterogeneity [11,12,13,14].

Despite their value in elucidating bladder cancer heterogeneity, transcriptome-based molecular classifications remain difficult to implement in routine clinical practice because of practical and technical constraints [9]. As a clinically feasible alternative, limited immunohistochemical marker panels have been proposed to recapitulate basal and luminal differentiation lineages consistent with transcriptome-defined subtypes [7,15,16]. However, the clinical implications of IHC-based molecular subtyping have yet to be comprehensively clarified. Accordingly, the present study investigated the associations between immunohistochemistry-based molecular subtypes, serving as practical surrogates of intrinsic differentiation lineages, and NAC response, pathological characteristics, as well as survival-related endpoints within a bladder cancer patient cohort.

## 2. Materials and Methods

### 2.1. Study Design and Patient Cohort

This single-center retrospective study consecutively enrolled patients with bladder cancer who received treatment at our institution between 17 May 2010 and 17 November 2025, and for whom complete clinical, pathological, and immunohistochemical data were available. Patients were eligible for inclusion only if they fulfilled all of the following criteria: histologically confirmed bladder cancer, complete clinicopathological information, and available immunohistochemical staining results for CK20, GATA3, CK5/6, and CK14 that permitted molecular subtype classification. Patients who failed to meet any of these criteria were excluded from the study. A total of 1507 patients were initially screened, and 590 patients were ultimately included in the final analysis after the exclusion of 917 patients with incomplete data. All patients underwent surgical treatment at our institution, and relevant clinical information was retrospectively collected from the hospital database. The patient selection process is summarized in the study flowchart.

### 2.2. Clinical Data Collection and Treatment Information

Clinical and pathological information was retrospectively retrieved from electronic medical records, including demographic characteristics, tumor-related clinicopathological features, and treatment information. Collected variables included age, sex, tumor stage, tumor grade, treatment modality, and neoadjuvant therapy status. Pathological staging and grading were performed based on the Tumor–Node–Metastasis (TNM) staging framework and established histopathological criteria. Tumor staging was assigned according to the American Joint Committee on Cancer (AJCC) staging system. Information on surgical management and neoadjuvant treatment, when applicable, was retrieved prior to outcome assessment. For the neoadjuvant therapy analysis, patients with clinically non-metastatic muscle-invasive bladder cancer (cT2–T4a, N0–3, M0) who received neoadjuvant systemic treatment followed by radical cystectomy (RC) were included. All patients underwent diagnostic and staging transurethral resection of bladder tumor (TURBT) before the initiation of systemic therapy. Neoadjuvant treatment primarily consisted of gemcitabine plus cisplatin (GC)-based regimens, although a minority of patients received combination treatment strategies according to contemporary clinical practice. Pathological response was evaluated on the final RC specimens. Pathological complete response (pCR) was defined as ypT0N0, and pathological downstaging was defined as a reduction in pathological stage compared with the pretreatment clinical stage.

### 2.3. Pathological Evaluation

Hematoxylin–eosin (H&E)-stained sections were independently reviewed by two experienced pathologists who were blinded to clinical outcomes. pCR and tumor downstaging were defined according to established criteria [17,18,19]. Additional histopathological features, including tumor budding, tumor–stroma proportion, and levels of tumor-infiltrating lymphocytes, were assessed using previously reported evaluation systems [20,21,22], according to previously published and widely accepted histopathological evaluation criteria. Tumor budding was evaluated at the invasive front and categorized as absent, low, intermediate, or high. Growth pattern was classified as cohesive, trabecular/nested, or spindle/single according to the predominant invasive morphology. Spreading pattern was categorized as compact or disseminating based on the overall pattern of tumor infiltration. Tertiary lymphoid structures (TLSs) were assessed as absent or present. Tumor–stroma ratio (TSR) was evaluated on representative tumor sections and categorized as low or high according to the relative proportion of stromal tissue within the tumor area. Stromal tumor-infiltrating lymphocytes (sTILs) were evaluated within the intratumoral stromal compartment and classified as absent, low, intermediate, or high according to the extent of lymphocytic infiltration. In cases of disagreement between the two pathologists, a consensus was reached through joint review.

### 2.4. Immunohistochemistry and Molecular Subtyping

Immunohistochemical staining was performed on formalin-fixed, paraffin-embedded (FFPE) tissue sections using clinically validated diagnostic antibodies according to the manufacturer’s instructions. The antibodies used for molecular subtyping included CK20, GATA3, CK5/6, and CK14. All staining procedures were performed on an automated immunohistochemical platform in the Department of Pathology of our institution following routine quality-control procedures.

Immunohistochemical slides were independently evaluated by two experienced genitourinary pathologists who were blinded to clinical information, treatment status, and patient outcomes. For each marker, staining intensity was assessed using a semi-quantitative scoring system (0, 1+, 2+, and 3+). Consistent with previously published immunohistochemistry-based molecular subtyping studies of bladder cancer, scores of 1+ to 3+ were considered positive, whereas a score of 0 was considered negative. For molecular subtype assignment, marker positivity was determined using a predefined staining intensity-based criterion rather than a quantitative scoring approach incorporating the proportion of positively stained tumor cells. Marker positivity was determined based on staining intensity, and the proportion of positively stained tumor cells was not incorporated into molecular subtype classification. Cases with discrepant interpretations were jointly reviewed until a consensus diagnosis was reached.

Molecular subtypes were assigned according to the expression patterns of luminal and basal markers. Tumors positive for CK20 and GATA3 but negative for CK5/6 and CK14 were classified as luminal subtype. Tumors positive for CK5/6 and/or CK14 but negative for CK20 were classified as basal subtype. Tumors expressing both luminal and basal markers were classified as double-positive subtype, whereas tumors lacking expression of both luminal and basal markers were classified as double-negative subtype. For the primary analyses, basal, double-positive, and double-negative tumors were grouped as non-luminal tumors, while additional analyses based on the original four-subtype classification were performed as sensitivity analyses.

### 2.5. Outcome Definitions and Follow-Up

Overall survival (OS) was calculated from the date of surgery to death from any cause. Recurrence-free survival (RFS) was defined as the time from surgery to the first documented tumor recurrence or death, whichever occurred first. Progression-free survival (PFS) was measured from surgery until documented disease progression or death. Patients without events were censored at the date of last follow-up. Follow-up information was obtained from medical records and routine clinical visits. The last follow-up date was used as the censoring time for patients who were alive and event-free. The median follow-up duration was calculated using the reverse Kaplan–Meier method. OS was considered the primary endpoint, whereas RFS and PFS were considered secondary endpoints.

### 2.6. Statistical Analysis

Statistical analyses were performed using SPSS software (version 25.0; IBM Corp., Armonk, NY, USA). Normality of continuous variables was assessed using the Shapiro–Wilk test in conjunction with visual inspection of Q–Q plots. Categorical variables were compared using the chi-square test or Fisher’s exact test, as appropriate. Continuous variables were expressed as mean ± standard deviation or median (interquartile range) depending on data distribution. Survival outcomes were derived using the Kaplan–Meier approach and subsequently compared via the log-rank test. Multivariable Cox proportional hazards regression models were applied to evaluate factors associated with survival outcomes. Hazard ratios (HRs) and 95% confidence intervals (CIs) were calculated. Variables included in the multivariable Cox regression models were selected based on their established clinical relevance and potential prognostic value in bladder cancer. To avoid redundancy and potential multicollinearity, highly correlated variables were not simultaneously included in the final model. Multicollinearity was assessed using variance inflation factors (VIFs), and proportional hazards assumptions were evaluated using log-minus-log survival plots. All statistical tests were two-sided, and a *p* value < 0.05 was considered statistically significant. Cases with missing data were excluded from the corresponding analyses. Graphs were generated using GraphPad Prism (version 10.0.2).

## 3. Results

### 3.1. Patient Characteristics

**Figure 1:**
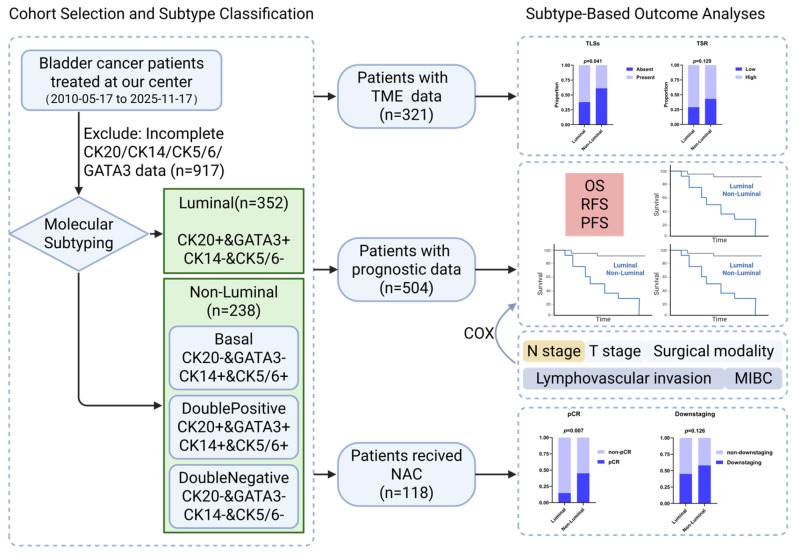
Patient cohort and study design.

Table 1 summarized the baseline clinical and pathological characteristics of the study population. A total of 590 patients constituted the final analytical cohort, as illustrated in Figure 1, of whom 352 (59.7%) were classified as luminal subtype and 238 (40.3%) as non-luminal subtype. The overall cohort had a mean age of 60 ± 19 years. Patients in the luminal and non-luminal groups had comparable ages (59 ± 20 vs. 62 ± 16 years, respectively). The majority of patients were male, accounting for 528 (89.5%) of the entire cohort, with similar proportions observed in the luminal and non-luminal groups (90.1% vs. 88.7%, *p* = 1.000). With respect to pathological tumor stage, the distribution of pathological T stage (Ta–T4) showed no statistically meaningful difference between the two molecular subtypes (*p* = 0.094). In the luminal group, Ta, T1, T2, T3, and T4 tumors accounted for 23.3%, 39.2%, 19.0%, 8.0%, and 7.7%, respectively, whereas the corresponding proportions in the non-luminal group were 16.8%, 13.9%, 17.2%, 34.0%, and 10.5%. Although the overall distribution did not reach statistical significance, non-luminal tumors tended to present with relatively higher proportions of advanced T-stage disease. Pathological nodal status was also comparable, with node-positive disease (≥N1) observed in 2.6% of luminal tumors and 3.4% of non-luminal tumors (*p* = 0.922).

Compared with non-luminal tumors, luminal tumors were significantly less likely to present with muscle-invasive disease. MIBC was observed in 37.5% of luminal tumors, whereas a substantially higher proportion of non-luminal tumors was muscle-invasive (69.3%, *p* < 0.001). Consistent with this distribution, patients with luminal tumors underwent RC less frequently than those with non-luminal tumors (44.0% vs. 72.7%, *p* = 0.001). With respect to pathological grade, luminal and non-luminal tumors were predominantly high grade, accounting for 88.4% and 90.3% of cases, respectively, with no significant difference observed between the two subtypes (*p* = 1.000).

The median follow-up duration for the entire cohort was 24.5 months (interquartile range [IQR], 13.0–39.0 months), and follow-up time did not differ significantly between the luminal and non-luminal groups (23.0 vs. 27.0 months, *p* = 0.140).

To assess potential selection bias resulting from the exclusion of patients with incomplete immunohistochemical data, baseline clinicopathological characteristics were compared between included and excluded patients. No significant differences were observed with respect to age, sex, pathological T stage, pathological N stage, tumor grade, MIBC status, or surgical modality (all *p* > 0.05), suggesting that patient exclusion was unlikely to introduce substantial selection bias (Supplementary Table S3).

### 3.2. Distinct Histopathological Patterns Between Luminal and Non-Luminal Bladder Cancers

**Figure 2:**
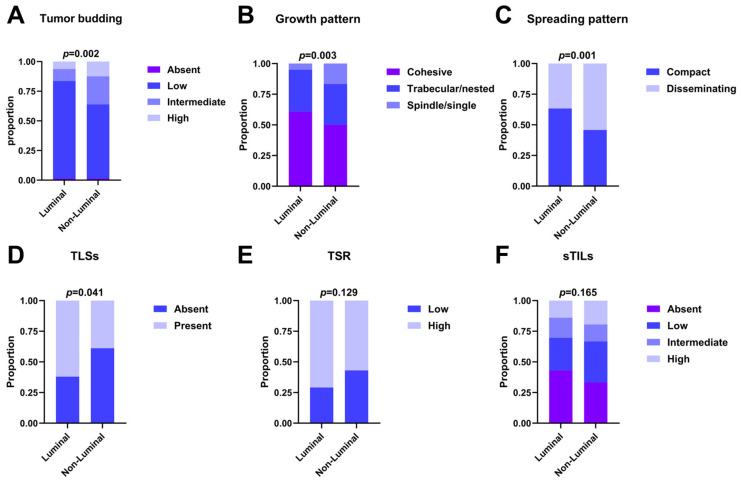
Pathological growth patterns and immune-related features according to molecular subtype. (**A**) Comparison of tumor budding grades between luminal and non-luminal tumors. (**B**) Comparison of predominant tumor growth patterns, including cohesive/trabecular–nested and non-cohesive or spindle/single-cell growth. (**C**) Comparison of tumor spreading patterns between molecular subtypes. (**D**) Comparison of tertiary lymphoid structure (TLS) status between luminal and non-luminal tumors. (**E**) Comparison of tumor–stroma ratio (TSR) between molecular subtypes. (**F**) Comparison of stromal tumor-infiltrating lymphocytes (sTILs) between luminal and non-luminal tumors.

At the morphological level, luminal tumors were less likely to exhibit aggressive growth characteristics than non-luminal tumors. Tumor budding was less prevalent in luminal tumors, with a lower proportion of intermediate- to high-grade budding (Figure 2A, *p* = 0.002). Regarding growth pattern, luminal tumors more commonly displayed cohesive or trabecular/nested architecture, whereas non-luminal tumors more frequently showed non-cohesive or spindle/single-cell growth (Figure 2B, *p* = 0.003). In addition, luminal tumors less frequently exhibited a disseminating spreading pattern compared with non-luminal tumors (Figure 2C, *p* = 0.001). Taken together, these observations indicate that luminal tumors tend to maintain more organized architectural growth patterns, whereas non-luminal tumors more frequently display infiltrative and dispersed growth behaviors.

With respect to immune-related pathological features, luminal tumors more frequently exhibited a higher frequency of TLSs, whereas the absence of TLSs was more common in non-luminal tumors (Figure 2D, *p* = 0.041). In contrast, no statistically meaningful differences were identified between luminal and non-luminal tumors in TSR (Figure 2E, *p* = 0.129) or sTILs (Figure 2F, *p* = 0.165). These findings suggest that subtype-related differences in the tumor microenvironment may be more prominently reflected by the presence of organized immune structures such as TLSs rather than by overall stromal composition or lymphocyte density.

### 3.3. NAC Response According to Molecular Subtypes

**Figure 3:**
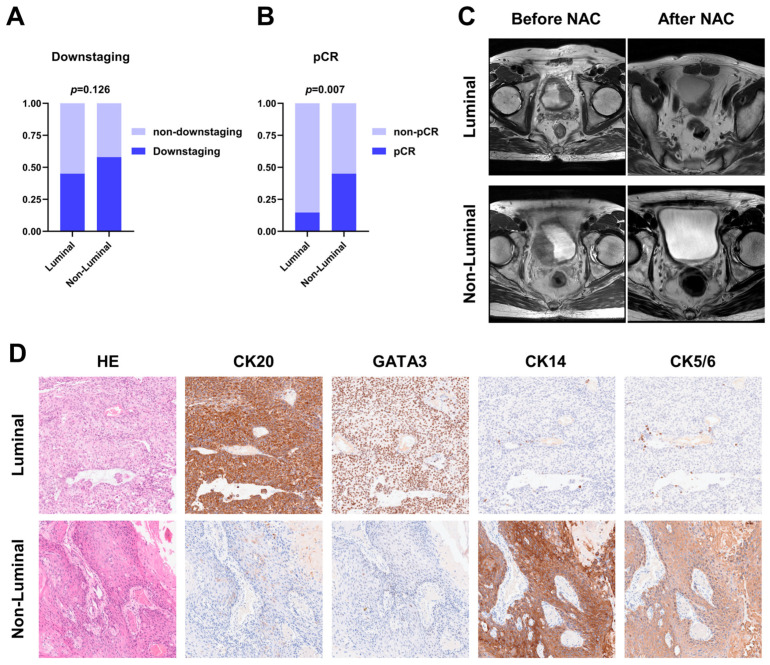
Neoadjuvant chemotherapy (NAC) response according to molecular subtypes. (**A**) Comparison of pathological downstaging rates between luminal and non-luminal tumors following NAC. (**B**) Comparison of pathological complete response (pCR) rates between luminal and non-luminal tumors. (**C**) Representative magnetic resonance imaging (MRI) scans illustrating radiological tumor appearance before and after NAC (NAC) in luminal and non-luminal bladder cancer patients. (**D**) Representative hematoxylin and eosin (H&E) and immunohistochemical staining images illustrating molecular subtype–specific histopathological and staining patterns in luminal and non-luminal tumors.

Among the entire cohort, 118 patients received NAC, including patients with both luminal and non-luminal tumors, Pathological response profiles differed between molecular subtypes (Figure 3). Luminal tumors demonstrated a reduced rate of pathological downstaging compared with non-luminal tumors. However, the observed difference was not statistically significant (Figure 3A, *p* = 0.126). Notably, this difference became more pronounced when pCR was considered. Non-luminal tumors achieved a significantly higher pCR rate compared with luminal tumors (Figure 3B, *p* = 0.007). Together, these findings indicated that luminal tumors were associated with inferior pathological responses to NAC relative to non-luminal tumors. These findings support a potential association between molecular subtype and pathological response to NAC in bladder cancer.

Figure 3C,D showed representative MRI images before and after NAC, together with corresponding IHC features, illustrating radiological and pathological characteristics of luminal and non-luminal tumors in patients receiving NAC.

### 3.4. Independent Prognostic Value of Molecular Subtypes for OS

**Figure 4:**
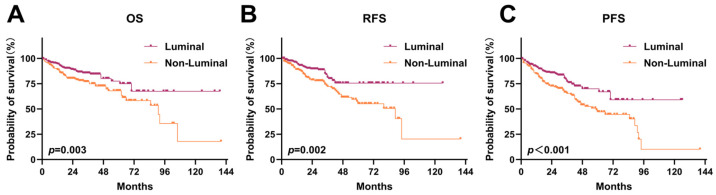
Survival outcomes according to molecular subtypes. Kaplan–Meier curves for overall survival (OS) (**A**), recurrence-free survival (RFS) (**B**), and progression-free survival (PFS) (**C**) comparing luminal and non-luminal bladder cancers. *p* values were calculated using the log-rank test.

To further evaluate the independent prognostic significance of molecular subtypes, Cox proportional hazards regression analyses were conducted on OS. Kaplan–Meier survival analyses demonstrated that patients with the luminal subtype exhibited significantly better survival outcomes compared with those with the non- across OS, RFS, and PFS (Figure 4A–C). Given the potential association between molecular subtype and clinicopathological characteristics, multivariable analysis was performed to adjust for potential confounding factors. Variables incorporated into the multivariable model included molecular subtype, MIBC status, pathological N stage, tumor grade, lymphovascular invasion, surgical modality, age, and gender.

Within the multivariable framework, molecular subtype was identified as an independent prognostic determinant for OS. Specifically, the luminal subtype functioned as a protective factor, with a lower hazard of death than the non-luminal subtype (HR = 0.51, 95% CI = 0.33–0.79, *p* = 0.003; Table 2).

In addition to molecular subtype, pathological N stage was identified as an independent predictor of OS (HR = 1.98, 95% CI = 1.14–3.42, *p* = 0.015). Other clinicopathological variables failed to retain independent significance after adjustment. These findings further support the independent prognostic relevance of molecular subtype beyond conventional clinicopathological factors.

Consistent with the OS analysis, multivariable Cox regression analyses for RFS and PFS showed that the luminal subtype was associated with a lower hazard of progression (HR = 0.55, 95% CI = 0.34–0.90, *p* = 0.017) and recurrence (HR = 0.44, 95% CI = 0.25–0.76, *p* = 0.003; Supplementary Tables S1 and S2).

To evaluate whether cohort heterogeneity influenced the prognostic value of molecular subtype, we performed additional stratified survival analyses according to disease status (NMIBC vs. MIBC) and surgical treatment (TURBT vs. RC). In both stratification schemes, patients with luminal tumors generally showed better survival outcomes than those with non-luminal tumors. This association was most pronounced in the MIBC and RC subgroups, while similar trends were observed in the NMIBC and TURBT subgroups. Although some subgroup analyses did not reach statistical significance, likely because of the smaller sample sizes after stratification, the overall findings remained consistent with those of the primary analysis. Detailed results are provided in Supplementary Figure S1. To further explore the potential biological heterogeneity within the non-luminal category, the original four-subtype classification (luminal, basal, double-positive, and double-negative) was reanalyzed. The distribution of the four immunohistochemistry-defined subtypes is summarized in Supplementary Table S4. Survival analyses based on the four-subtype classification demonstrated significant differences in OS, RFS, and PFS among the subgroups (log-rank *p* = 0.033, 0.010, and 0.003, respectively; Supplementary Figure S2A–C). Additional pairwise comparisons showed that luminal tumors generally exhibited more favorable survival outcomes than basal, double-positive, and double-negative tumors (Supplementary Figure S2D–L). These findings support the robustness of the primary luminal versus non-luminal classification while also highlighting the biological heterogeneity within the non-luminal category.

Model diagnostics were subsequently performed for the multivariable Cox regression analyses. All VIF values were below 1.5, indicating no evidence of substantial multicollinearity among the included variables (Supplementary Table S5). In addition, log-minus-log survival plots demonstrated approximately parallel curves without substantial crossing, supporting the proportional hazards assumption for the Cox regression models (Supplementary Figure S3).

## 4. Discussion

Within this retrospective cohort, we evaluated the clinical relevance of an immunohistochemistry-based molecular classification in bladder cancer using routinely available pathological markers. Specifically, non-luminal tumors were more prone to attaining pCR following NAC, whereas luminal tumors demonstrated more favorable long-term survival. At the pathological level, non-luminal tumors exhibited more aggressive features compared with luminal tumors. Together, these observations suggest that simplified immunophenotypic classification can capture clinically meaningful biological differences that parallel those identified in transcriptome-based molecular taxonomies.

Bladder cancer represents a highly heterogeneous malignancy in which patients with apparently similar clinicopathological characteristics may experience markedly different therapeutic responses and survival outcomes [5]. In recent years, molecular subtyping has emerged as a key framework for stratifying this biological heterogeneity and for linking tumor biology with treatment response and prognosis [6,7,8,9]. Prior work has established that genes such as GATA3 and KRT20 are closely associated with the maintenance of urothelial differentiation and structural stability, thereby enabling luminal tumors to retain favorable cell–cell adhesion characteristics. These markers are widely used as luminal molecular phenotypes associated with urothelial lineage differentiation and epithelial organization, which represent defining features of luminal subtype described in transcriptome-based classification systems [7,9,12]. In contrast, non-luminal tumors, defined by expression patterns involving KRT5/6 and KRT14, represent a less differentiated and more structurally plastic cellular state [8], which has been associated with increased proliferative and invasive growth characteristics and may underlie their propensity to exhibit tumor budding, non-cohesive or single-cell growth, and disseminated invasive patterns [7,12,21,22]. Consistent with these observations, our immunohistochemistry-based classification demonstrated that non-luminal tumors more frequently exhibited increased tumor budding, non-cohesive or spindle/single-cell growth, and disseminated spreading patterns, whereas luminal tumors retained more cohesive and organized architectural features. These findings indicate that simplified immunophenotypic subtyping delineates biologically distinct tumor growth patterns with recognizable histomorphological features.

Molecular subtype-specific differences in response to NAC have been increasingly recognized in bladder cancer. Neoadjuvant cisplatin-based chemotherapy has been established as a considerably important component of treatment for muscle-invasive bladder cancer, improving survival when administered prior to RC [19]. However, the magnitude of benefit varies substantially across patients, highlighting the need for biological markers capable of predicting therapeutic response. Prior transcriptome-based studies consistently reported that basal or basal/squamous tumors exhibit greater chemosensitivity to cisplatin-based NAC than luminal tumors [9,23]. In line with these observations, our immunohistochemistry-based classification similarly demonstrated that non-luminal tumors achieved a significantly higher pCR rate following NAC, supporting the ability to reflect of simplified immunophenotypic subtyping in capturing clinically relevant treatment response patterns. Further studies have suggested that the enhanced chemosensitivity of basal or non-luminal bladder cancers is related to higher proliferative activity and lower differentiation, features that are typically associated with accelerated cell-cycle progression and increased replication stress, thereby rendering tumor cells more vulnerable to cytotoxic agents that induce DNA damage [24]. In addition, basal-like tumors exhibit distinct transcriptional programs involving DNA damage response pathways [23], reflecting a cellular state that is highly dependent on efficient DNA repair mechanisms to maintain genomic integrity. Consistent with this, molecular classifications based on large-scale consensus and TCGA datasets have shown that basal-like tumors are characterized by increased genomic instability and elevated proliferative indices [9], biological features that further amplify the accumulation of chemotherapy-induced DNA lesions. Under such conditions, the capacity of tumor cells to tolerate DNA damage becomes limited, which may enhance susceptibility to DNA-damaging agents such as cisplatin and contribute to improved pathological responses following NAC [9,12]. Together, these findings provide a biological rationale for the observed association between immunohistochemistry-based subtypes and differential response to NAC. Nevertheless, the present NAC analysis should be interpreted with caution because of its retrospective nature, limited sample size, and lack of multivariable adjustment. Therefore, our findings support an association between molecular subtype and pathological response rather than establishing molecular subtype as a definitive predictive biomarker. Future prospective studies with larger cohorts are warranted to further validate these observations.

Recent studies have demonstrated that molecular subtyping provides prognostic information in bladder cancer, with transcriptome-based classifications consistently showing that luminal tumors achieve more favorable overall and disease-specific survival, whereas basal tumors experience inferior long-term outcomes, a pattern further supported by meta-analyses across independent cohorts [9,10]. Our findings are fully concordant with this evidence, as patients with luminal tumors exhibited superior overall, recurrence-free, and progression-free survival compared with those with non-luminal tumors. Integrating our histopathological analyses, this survival advantage appears to be driven by distinct pathological growth characteristics: luminal tumors more frequently displayed cohesive and organized architecture and structured immune-related features, whereas non-luminal tumors showed aggressive microscopic behavior, including increased tumor budding, non-cohesive or spindle/single-cell growth, and disseminated spreading patterns. These aggressive growth patterns have been linked to increased invasive potential and adverse oncologic outcomes in several solid tumors, including urothelial carcinoma [21,22]. Consistent with prior transcriptome-based and pathological studies, these features reflect fundamental biological differences between molecular subtypes. Luminal tumors generally maintain a higher degree of differentiation and exhibit a more indolent clinical course, whereas basal or non-luminal tumors are more frequently associated with dedifferentiation, infiltrative growth, and increased proliferative activity [9,10]. Although non-luminal tumors achieved higher pCR rates following NAC, this apparent discrepancy did not translate into superior long-term survival outcomes in our cohort. Several factors may contribute to this observation, including differences in baseline tumor aggressiveness, stage distribution, residual heterogeneity within the non-luminal category, and other unmeasured confounding factors inherent to retrospective analyses. Therefore, these findings should be interpreted as an observed association rather than definitive evidence of a biological dissociation between treatment response and long-term prognosis. From a translational perspective, an important implication of the present findings lies in the feasibility of implementing molecular stratification in routine pathology practice. While transcriptome-based classification systems have provided important biological insights, their widespread clinical adoption remains limited by cost, technical requirements, and turnaround time [5,15]. In contrast, immunohistochemistry-based panels using markers such as GATA3, CK20, CK5/6, and CK14 have been proposed as practical surrogates capable of approximating luminal and basal molecular phenotypes in clinical settings [10,25]. Our results further support this concept by demonstrating that a simplified IHC-based classification can recapitulate key biological and clinical characteristics previously described in sequencing-based molecular subtypes.

Several limitations of this study should be acknowledged. First, molecular subtyping was inferred using an immunohistochemistry-based panel rather than comprehensive transcriptomic profiling. While this approach improves feasibility and clinical translatability, the concordance between IHC-based classification and sequencing-based molecular subtypes warrants further validation. Second, although the primary analyses were performed using a clinically practical luminal versus non-luminal framework, we acknowledge the biological heterogeneity within the non-luminal category. To address this issue, additional analyses based on the original four-subtype classification were performed. The overall findings remained generally consistent, supporting the prognostic relevance of the proposed classification system while underscoring the complexity of molecular subtype heterogeneity in bladder cancer. Given the well-recognized biological heterogeneity inherent to bladder cancer molecular subtypes reported in previous studies [10], the extent of within-group biological consistency merits further investigation. Future studies integrating multi-omics data may help further refine the biological heterogeneity that exists within these broad phenotypic categories. Third, although the present findings were derived from a retrospective cohort, prospective validation in independent cohorts would further strengthen the generalizability of these observations. In addition, incorporating molecular subtyping into prospective treatment stratification trials may help clarify whether subtype-guided therapeutic strategies can further improve clinical outcomes.

Together, these findings indicate that IHC-based molecular subtyping delineates biologically distinct molecular categories of bladder cancer characterized by consistent differences in pathological characteristics, treatment response, and survival outcomes. By bridging molecular subtype identity with observable histopathological features and clinically relevant endpoints, this simplified classification framework may facilitate the integration of molecular biology into routine pathological evaluation and clinical decision-making in bladder cancer.

## Figures and Tables

**Table 1:** Clinical characteristics of patients across the groups.

Characteristics	Overall	Luminal	Non-Luminal	*p* Value
Frequency	590	352	238	
Age, mean (SD)	60 ± 19	59 ± 20	62 ± 16	0.397
Gender (%) Male Female	528 (89.5%) 62 (10.5%)	317 (90.1%) 35 (9.9%)	211 (88.7%) 27 (11.3%)	1.000
Pathological T stage (%) Ta T1 T2 T3 T4	122 (20.7%) 171 (29.0%) 108 (18.3%) 109 (18.5%) 52 (8.8%)	82 (23.3%) 138 (39.2%) 67 (19.0%) 28 (8.0%) 27 (7.7%)	40 (16.8%) 33 (13.9%) 41 (17.2%) 81 (34.0%) 25 (10.5%)	0.094
Pathological N stage (%) N0/Nx ≥N1	573 (97.1%) 17 (2.9%)	343 (97.4%) 9 (2.6%)	230 (96.6%) 8 (3.4%)	0.922
Pathological grade (%) Low High	64 (10.8%) 526 (89.2%)	41 (11.6%) 311 (88.4%)	23 (9.7%) 215 (90.3%)	1.000
MIBC Absent Present	293 (49.7%) 297 (50.3%)	220 (62.5%) 132 (37.5%)	73 (30.7%) 165 (69.3%)	0.001
Surgery modalities (%) TURBT radical cystectomy	262 (44.4%) 328 (55.6%)	197 (56.0%) 155 (44.0%)	65 (27.3%) 173 (72.7%)	0.001
OS, months (Q1, Q3)	24.5 (13.0, 39.0)	23.0 (13.0, 37.25)	27.0 (13.0, 48.75)	0.140

**Table 2:** Multivariable Cox regression analysis of factors associated with OS.

Characteristics	HR	95% CI	*p* Value
Molecular subtype			
Non-Luminal	1.00	Reference	
Luminal	0.51	0.33–0.79	0.003
MIBC			
No	1.00	Reference	
Yes	1.15	0.59–2.23	0.678
Pathological N stage			
N0	1.00	Reference	
N+	1.98	1.14–3.42	0.015
Tumor grade			
Low grade	1.00	Reference	
High grade	2.44	0.58–10.26	0.225
Lymphovascular invasion			
No	1.00	Reference	
Yes	1.28	0.76–2.16	0.349
Surgical modality			
TURBT	1.00	Reference	
Radical cystectomy	1.38	0.70–2.71	0.348
Age			
Per year increase	1.01	0.99–1.03	0.323
Gender			
Male	1.00	Reference	
Female	1.79	0.92–3.49	0.086

## Data Availability

All data generated or analyzed during this study are included in this published article and its Supplementary Information Files. Further enquiries can be directed to the corresponding authors.

## Supplementary Materials

The following supporting information can be downloaded at: https://www.mdpi.com/article/10.3390/diagnostics16132055/s1, Figure S1: Kaplan-Meier survival analyses stratified by molecular subtype in different clinical subgroups; Figure S2: Kaplan-Meier survival analyses comparing molecular subtypes defined by immunohistochemistry; Figure S3: Log-minus-log survival plots for assessing the proportional hazards assumption; Table S1: Multivariable Cox regression analysis of factors associated with progression-free survival; Table S2: Multivariable Cox regression analysis of factors associated with recurrence-free survival; Table S3: Comparison of baseline clinicopathological characteristics between included and excluded patients; Table S4: Distribution of the four immunohistochemistry-based molecular subtypes; Table S5: Multicollinearity assessment of variables included in the multivariable Cox regression model.
