# Supplementary material for: An Immunohistochemistry-Based Molecular Subtyping Approach for Capturing Clinical Outcome Heterogeneity in Bladder Cancer
Source: Diagnostics (Basel). 2026 Jun 30;16(13):2055. doi: 10.3390/diagnostics16132055 (PMC13360000; doi:10.3390/diagnostics16132055)
Supplement: Supplementary file 1 [file diagnostics-16-02055-s001.zip › diagnostics-4237802-supplementary.pdf]

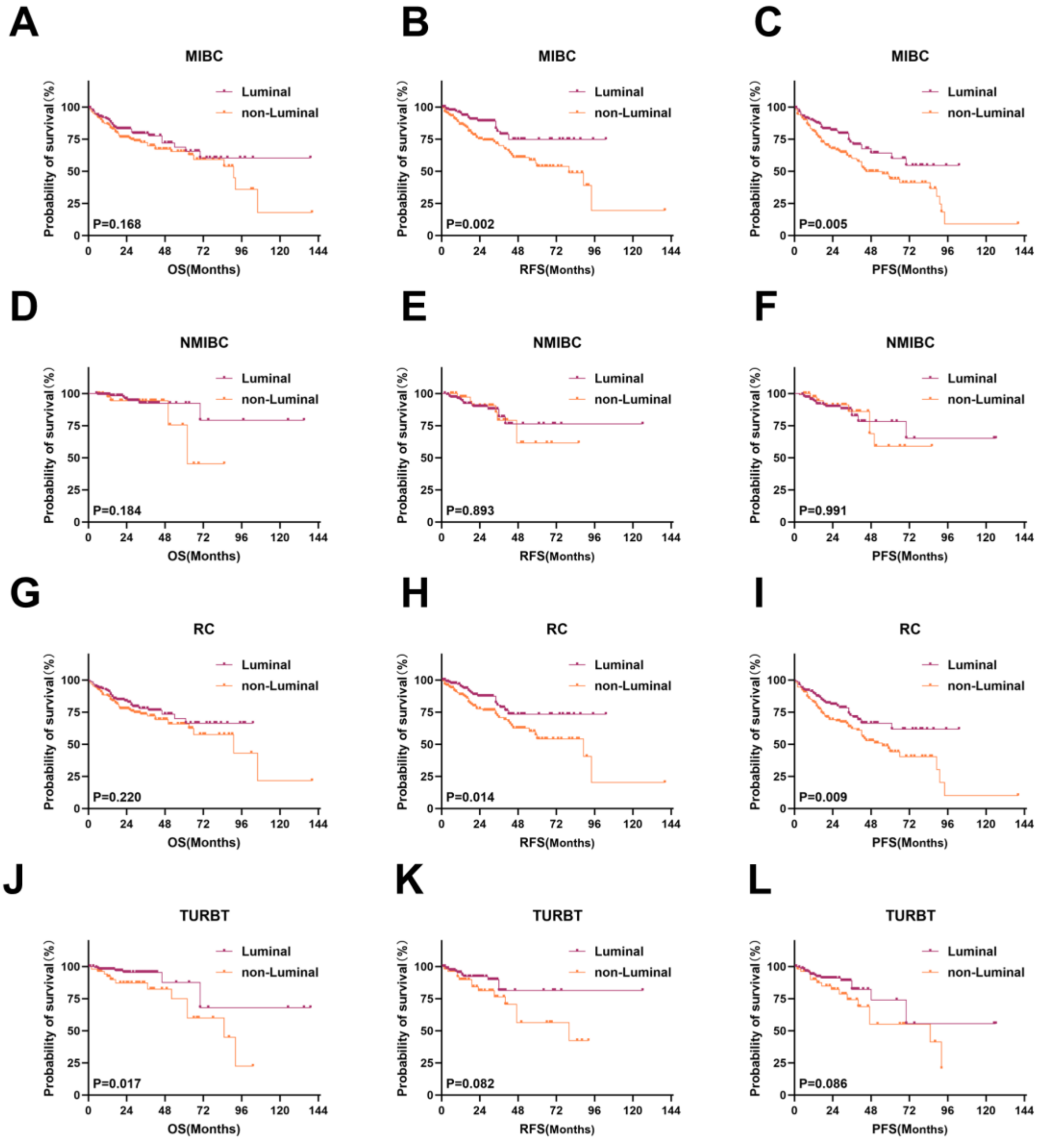

Figure S1. Kaplan-Meier survival analyses stratified by molecular subtype in different clinical subgroups.

(A–C) Overall survival (OS), recurrence-free survival (RFS), and progression-free survival (PFS) in patients with muscle-invasive bladder cancer (MIBC).

(D–F) OS, RFS, and PFS in patients with non-muscle-invasive bladder cancer (NMIBC).

(G–I) OS, RFS, and PFS in patients treated with radical cystectomy (RC).

(J–L) OS, RFS, and PFS in patients treated with transurethral resection of bladder tumor (TURBT).

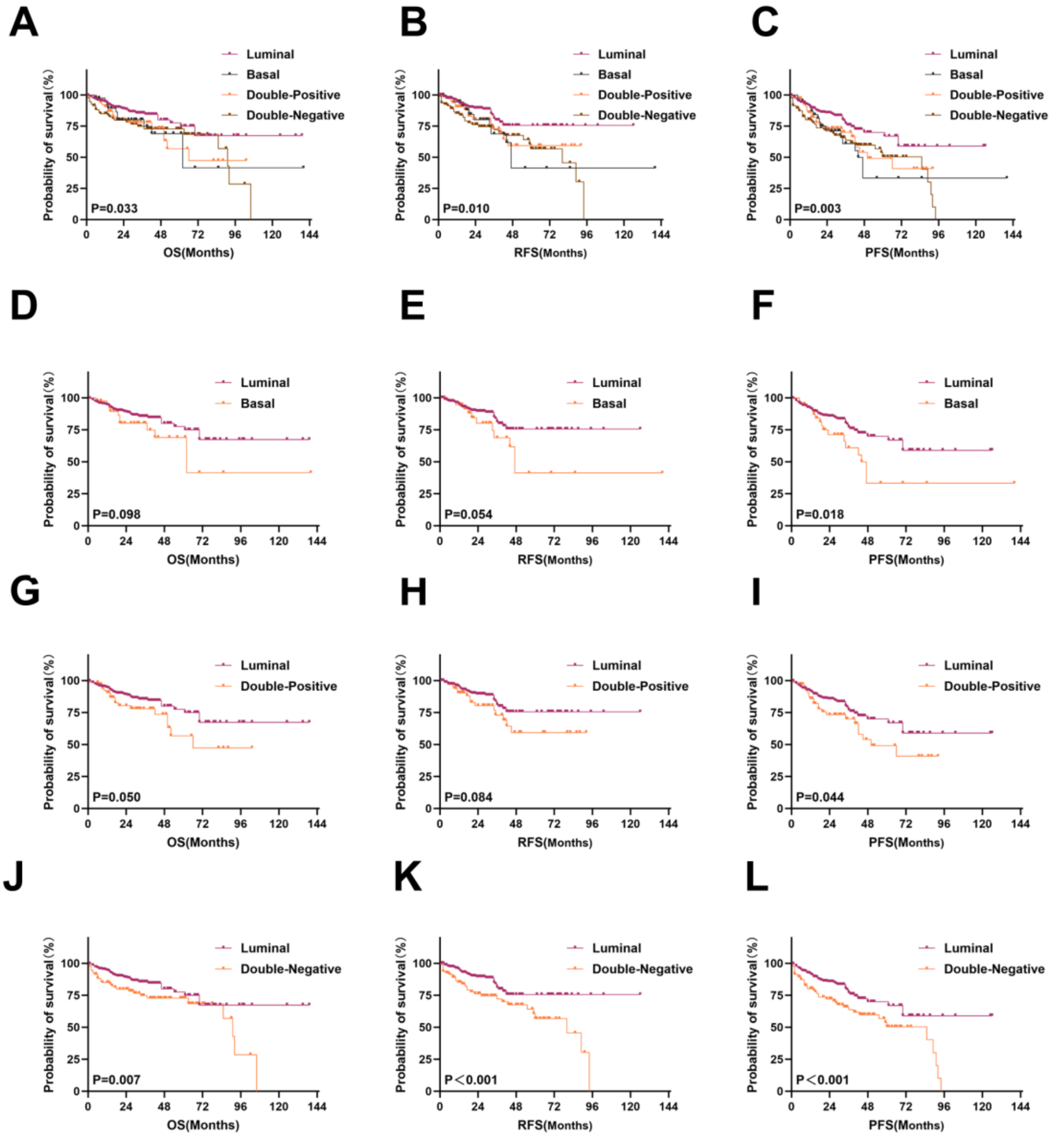

Figure S2. Kaplan-Meier survival analyses comparing molecular subtypes defined by immunohistochemistry.

(A–C) Overall survival (OS), recurrence-free survival (RFS), and progression-free survival (PFS) among luminal, basal, double-positive, and double-negative subtypes.

(D–F) OS, RFS, and PFS comparing luminal and basal subtypes.

(G–I) OS, RFS, and PFS comparing luminal and double-positive subtypes.

(J–L) OS, RFS, and PFS comparing luminal and double-negative subtypes.

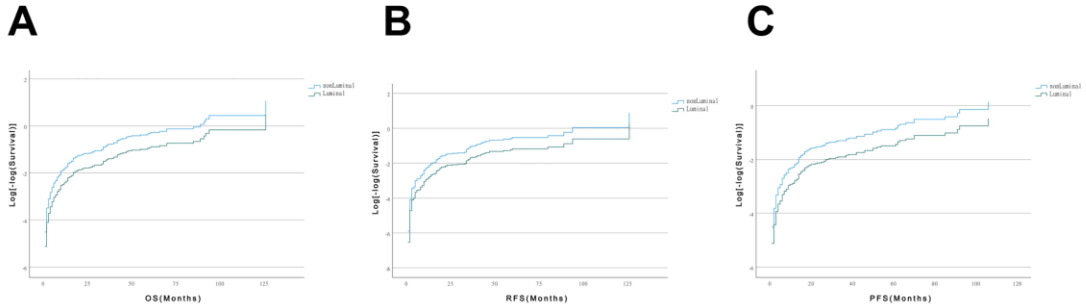

Figure S3. Log-minus-log survival plots were generated to assess the proportional hazards assumption for the comparison between luminal and non-luminal subtypes.

(A) Log-minus-log plot for overall survival (OS) comparing luminal and non-luminal subtypes.

(B) Log-minus-log plot for recurrence-free survival (RFS) comparing luminal and non-luminal subtypes.

(C) Log-minus-log plot for progression-free survival (PFS) comparing luminal and non-luminal subtypes.

Supplementary Table S1. Multivariable Cox regression analysis of factors associated with progression-free survival

Characteristics	HR	95% CI[a]	P value[a]
**Molecular subtype**			
Non-Luminal	1.00	Reference	
Luminal	0.55	0.34–0.90	0.017
**MIBC**			
No	1.00	Reference	
Yes	1.15	0.54–2.45	0.713
**Pathological N stage**			
N0	1.00	Reference	
N+	3.24	1.82–5.78	<0.001
**Tumor grade**			
Low grade	1.00	Reference	
High grade	3.18	0.42–23.84	0.261
**Lymphovascular invasion**			
No	1.00	Reference	
Yes	1.28	0.72-2.27	0.397
**Surgical modality**			
TURBT	1.00	Reference	
Radical cystectomy	1.47	0.68–3.18	0.325
**Age**			
Per year increase[b]	1.01	0.99-1.03	0.300
**Gender**			
Male	1.00	Reference	
Female	1.75	0.83–3.68	0.140

a: Hazard ratios (HRs) and 95% confidence intervals (CIs) were estimated using a Cox proportional hazards model with all variables entered simultaneously.

b: Age were analyzed as continuous variables and are presented per 1-year increase, respectively.

Supplementary Table S2. Multivariable Cox regression analysis of factors associated with recurrence-free survival

Characteristics	HR	95% CI[a]	P value[a]
**Molecular subtype**			
Non-Luminal	1.00	Reference	
Luminal	0.44	0.25–0.76	0.003
**MIBC**			
No	1.00	Reference	
Yes	1.07	0.50–2.29	0.868
**Pathological N stage**			
N0	1.00	Reference	
N+	1.67	0.83–3.34	0.151
**Tumor grade**			
Low grade	1.00	Reference	
High grade	1.78	0.42–7.58	0.437
**Lymphovascular invasion**			
No	1.00	Reference	
Yes	1.46	0.77–2.74	0.245
**Surgical modality**			
TURBT	1.00	Reference	
Radical cystectomy	1.12	0.52–2.38	0.777
**Age**			
Per year increase[b]	1.02	1.00–1.04	0.088
**Gender**			
Male	1.00	Reference	
Female	1.91	0.86–4.26	0.113

a: Hazard ratios (HRs) and 95% confidence intervals (CIs) were estimated using a Cox proportional hazards model with all variables entered simultaneously.

b: Age were analyzed as continuous variables and are presented per 1-year increase, respectively.

Supplementary Table S3. Comparison of baseline clinicopathological characteristics between included and excluded patients

Variable	Included patients	Excluded patients	P value
**Frequency**	590	917	
**Age, mean (SD)**	60 ± 19	62 ± 21	0.056
**Gender (%)**			0.780
Male	528 (89.5%)	826 (90.7%)	
Female	62 (10.5%)	91 (9.3%)	
**Pathological T stage (%)**			0.422
Ta	122 (20.7%)	178 (19.4%)	
T1	171 (29.0%)	275 (30.0%)	
T2	108 (18.3%)	174 (19.0%)	
T3	109 (18.5%)	177 (19.3%)	
T4	52 (8.8%)	113 (12.3%)	
**Pathological N stage (%)**			0.985
N0/Nx	573 (97.1%)	892 (97.3%)	
≥N1	17 (2.9%)	25 (2.7%)	
**Pathological grade (%)**			0.625
Low	64 (10.8%)	91 (9.9%)	
High	526 (89.2%)	826 (90.1%)	
**MIBC**			0.923
Absent	293 (49.7%)	459 (50.1%)	
Present	297 (50.3%)	458 (49.9%)	
**Surgery modalities (%)**			0.298
TURBT	262 (44.4%)	381 (41.5%)	
RC	328 (55.6%)	536 (58.5%)	

Supplementary Table S4. Distribution of the four immunohistochemistry-based molecular subtypes

Molecular subtype	n	%
**Luminal**	352	59.7
**Basal**	50	8.5
**Double-positive**	85	14.4
**Double-negative**	103	17.4
**Total**	590	100.0

Supplementary Table S5. Multicollinearity assessment of variables included in the multivariable Cox regression model[a]

Variable	Tolerance	VIF
**Molecular subtype**	0.897	1.115
**MIBC**	0.687	1.457
**Pathological N stage**	0.821	1.217
**Tumor grade**	0.885	1.130
**Lymphovascular invasion**	0.801	1.248
**Surgical modality**	0.718	1.393
**Age**	0.993	1.007
**Gender**	0.988	1.012

a: Tolerance and variance inflation factor (VIF) were used to assess multicollinearity.
